# Dynamics and Diversity of Microbial Contamination in Poultry Bedding Materials Containing Parts of Medicinal Plants

**DOI:** 10.3390/ma15041290

**Published:** 2022-02-09

**Authors:** Łukasz Gontar, Monika Sitarek-Andrzejczyk, Maksymilian Kochański, Maria Buła, Andżelika Drutowska, Dariusz Zych, Justyna Markiewicz

**Affiliations:** Research and Innovation Centre Pro-Akademia, Innowacyjna 9/11, 95-050 Konstantynow Lodzki, Poland; maksymilian.kochanski@proakademia.eu (M.K.); maria.bula@proakademia.eu (M.B.); andzelika.drutowska@proakademia.eu (A.D.); dariusz.zych@proakademia.eu (D.Z.); justyna.markiewicz@proakademia.eu (J.M.)

**Keywords:** poultry litter, pellets, broiler house, *Satureja hortensis*, *Origanum vulgare*, *Staphylococcus*, *Escherichia*, *Listeria*, *Salmonella*, *Candida*

## Abstract

Microorganisms thriving in poultry bedding materials during their exploitation are involved in the development of several diseases and disfunctions of animals. They can also contaminate food products and pose risks to the environment and human health. This study provides an analysis of dynamics and diversity in microbiological contamination observed during the exploitation of poultry bedding materials containing parts of medicinal plants: *Satureja hortensis, Origanum vulgare*, *Melissa officinalis*, *Salvia officinalis*, and *Thymus vulgaris*, compared with standard types of beddings: straw chaff and straw pellets. The research was carried out in two 42-day experimental cycles involving in total 2400 broiler chickens. Each week, the total count of mesophilic bacteria, fungi and yeasts, the presumptive presence and count of *Staphylococcus* sp., *Escherichia* sp., *Listeria* sp., *Salmonella* sp., and *Candida* sp. were determined by culturing on selective media, along with pH and moisture measurements. After 35 days of the experiment, a reduction of the total count of mesophilic bacteria above 1 log compared to the control (11.86 vs. 13.02 log CFU/g) was observed. As the count of yeasts decreased after 21 days, an increase in the total count of bacteria was reported, which indicates a strong competition between microorganisms. The results improve our understanding of the temporal effects of using materials containing parts of medicinal plants on the microbial contamination in poultry litter.

## 1. Introduction

Between 1961 and 2020, the global poultry production increased from 9 to 134 million tonnes [1,2]. Along with this growing supply and demand, a strong societal and regulatory pressure to improve the sustainability of animal production can be observed. For instance, in response to the European Citizens’ Initiative to ‘End the Cage Age’, in 2021 the European Commission announced its plan to prohibit the use of cages in the European Union from 2027 for all laying hens, broiler breeders, layer breeders, quail, ducks and geese [3]. These trends are important factors stimulating the demand for poultry bedding materials. During exploitation, these materials are mixed by animals with their excreta, spilt feed, and feathers. The resulting poultry litter hosts rich and diverse populations of microorganisms [4]. Several genera of pathogenic bacteria, fungi, and yeasts present in it are conducive to the development of secondary infections and disfunctions of animals, contamination of food products, and the environment. For instance, pathogenic *Staphylococcus* sp. have been associated with animal lameness, endocarditis, fibrinopurulent blepharitis, and conjunctivitis [5,6]. Genera of *Escherichia* sp. have been linked with colibacillosis, salpingitis peritonitis, omphalitis, enteritis, respiratory tract infection, and septicemia [7,8]. *Listeria* sp. can occur in both bedding materials and in poultry products, making them the primary food vehicles for listeriosis in humans [9]. Similar negative impacts concern *Salmonella* sp., whose transmission via contaminated eggs to humans is another international public health concern [10]. Pathogenic *Candida* sp. have been associated with candidiasis [11]. Furthermore, an incorrect use of antibiotics to fight pathogens may result in the development of antibiotic resistance in the animal’s gastrointestinal tract, which can be then transferred through the litter to arable land, causing wider damages in the environment [12]. Direct land application of chicken litter could be harming animal, human, and environmental health due to its high fecal bacteria microbial load [13].

In this context, ensuring appropriate materials for bedding is among the most important biosecurity practices in poultry production [14]. Sawdust, bark, straw, and peat have been traditionally used as poultry bedding materials for many years around the world [15]. Materials derived from recycled wood and paper industries, e.g., shredded paper, dried paper sludge, recycled cardboard, and wood shavings, have been also applied [16]. Another type of beddings are materials based on by-products of crop industries, e.g., cereal crop residues, crop and nut hulls [17]. Such feedstocks can be also pelletised. For instance, straw pellets are becoming a more and more popular bedding material for poultry [18]. This can be explained by the relatively high water absorption capacity and low dustiness of straw pellets as compared to the traditionally used materials, such as straw chaff and wood shavings.

Within the group of beddings based on by-products of crop industries, materials comprised of parts of medicinal plants or by-products from their harvesting, can be used as well [19]. They have an important advantage over other types of bedding materials, i.e., a potential biological activity. The activity of natural products from medicinal plants against microbial threats in poultry production has been widely documented, e.g., for *Satureja hortensis* L. [20], *Origanum vulgare* L. [21], *Melissa officinalis* L. [22], *Salvia officinalis* L. [23], and *Thymus vulgaris* L. [24]. Their activity has also been proven against several methicillin-resistant poultry bacteria threatening public health, such as *Staphylococcus aureus*, *Escherichia coli*, *Klebsiella pneumonia*, *Pseudomonas aeruginosa*, *Proteus mirabilis*, *Brevibacterium ammoniagenes*, and *Streptococcus mutans* [18]. Therefore, due to the high content of essential oils with proven antimicrobial properties, materials comprising parts of such plants are a promising natural option not only for improving on-farm biosecurity, but also fighting against antimicrobial-resistant microorganisms.

Even though the microbiological contamination of various used poultry bedding materials has been a subject of several prior studies [25,26,27,28,29,30,31,32,33,34,35], still little is known about the effects of using parts of different medicinal plants on the microbial contamination in bedding materials containing such parts. Given this background, the objective of our study is to investigate the total count of mesophilic aerobic bacteria, the total count of yeasts and moulds, and the presumptive count of *Staphylococcus* sp., *Escherichia* sp., *Listeria* sp., *Salmonella* sp., and *Candida* sp. in poultry bedding materials containing parts of *Satureja hortensis*, *Origanum vulgare*, *Melissa officinalis*, *Salvia officinalis*, and *Thymus vulgaris* during the exploitation of materials by animals.

The results improve our understanding of the temporal effects of using materials containing parts of medicinal plants on the microbial contamination in poultry litter.

## 2. Materials and Methods

### 2.1. Materials Production and Composition

Six prototypes of materials containing parts of medicinal plants (P1–P6), as well as two control materials (triticale straw pellet and chaff), were produced at Research and Innovation Centre Pro-Akademia (Konstantynów Łódzki, Poland). The semi-industrial pelleting line (Nawrocki Pelleting Technology, Żnin, Poland), equipped with the preliminary and regrinding mill, miniPelleter 18 granulator, and a vibratory cooling system, was used. In all materials, triticale straw constituted the main component (Table 1). One hundred kilograms of straw pellets and materials P1–P6 in the form of pellets, with a 6 ± 1 mm diameter and length between 10 and 35 mm, were obtained in pelleting process, which was as follows. After about 4–6 s of being in the working chamber (40–60 °C), mixed cut raw material (ingredients 1, 2, and 3) was pressed into the sieve-shaped matrix. There, during 3–4 cycles of denting with rollers in the matrix, it was exposed to elevated temperature (100 °C) and pressure (150 MPa). Then it was squeezed out of the matrix with successive layers of raw material to the output chamber and from there it was pushed onto the sifter with the scraper.

Additionally, 100 kg of triticale straw chaff was produced by milling. Subsequently, all produced materials were transported for experiments involving animals at the Agricultural Experimental Station Wilanów Obory (Warsaw University of Life Sciences, Poland) (see Section 2.3). Samples of materials for their physico-chemical and microbiological characterisation prior exploitation were collected as well.

### 2.2. Physico-Chemical and Microbiological Characterisation of Materials Prior to Their Exploitation

#### 2.2.1. Essential Oil Content

The essential oil content in the produced materials as well as in the input ingredients used for the production of materials was measured according to the Polish *Pharmacopoeia* (FP X). One hundred and fifty grams (±0.01 g) of air-dry mass of bedding materials were hydrodistilled for 3 h in a Clevenger apparatus. Similarly, 50 g (±0.01 g) of air-dry mass of input ingredients were hydrodistilled for 3 h in a Clevenger apparatus. The obtained volume of oil was read from the scale of the apparatus receiver. For each material and input ingredient, the analysis of the oil content was carried out in triplicate. The essential oil content was expressed in ml of essential oil per 100 g of the air-dry mass [ml/100 g a.d.m.].

#### 2.2.2. Moisture Content

The mean water content (c, [%]) in the bedding materials was determined according to the oven method (105 °C) [36].

#### 2.2.3. Water Absorption Capacity

The water absorption capacity of the materials was determined according to the following method. Forty grams of the bedding material was placed in a beaker and poured with 400 mL of distilled water. Then the sample was thoroughly mixed using a glass rod. After 30 min, the unabsorbed water from the sample was drained by placing it on a sieve with a mesh diameter of 0.8 mm. Twenty grams of the drained sample was placed in a weighing dish in an oven and dried for 24 h (105 °C). The water content in the soaked sample (s, [g]) was determined according to the oven method (105 °C) [36]. Then, the mass of the soaked sample after drying was weighed (b, [g]). After that, the mass of water absorbed by the sample (M) of bedding material was calculated according to the Formula (1):

M = s × (1 − c)(1)

Finally, the absorption capacity of the bedding material (A, [%]) was calculated according to the Formula (2):

A = M/b(2)

#### 2.2.4. pH Value

The pH value of materials was measured with a pH meter Lab 850 (SiAnalytics GmbH, Mainz, Germany). Its probe was inserted into 25 g of the bedding suspended in 300 mL of Maximum Recovery Diluent (Oxoid, Hampshire, UK). Next, the pH value was read.

#### 2.2.5. Total Count of Aerobic Mesophilic Bacteria

Samples of 25 g of each material were weighed in sterile filter bags (BagFilter P, Interscience, Saint Nom la Bretêche, France) containing 300 mL of Maximum Recovery Diluent (Oxoid, Hampshire, UK) and ground in a stomacher homogeniser (Bag Mixer 400 LW, Interscience, Saint Nom la Bretêche, France). Series of ten-fold dilutions were made from the suspension of bedding after grounding and inoculated directly on the surface of the appropriate selective medium by an automatic spiral plater (easySpiral^®^Pro, Interscience, Saint Nom la Bretêche, France). Each sample dilution (0.05 mL) was surface inoculated on tryptone soya agar (Oxoid, Hampshire, UK) for a total count of aerobic mesophilic bacteria and incubated at 30 °C for 48 h in an air-jacketed incubator (NuAire 5831, NuAire Inc., Plymouth, MN, USA). Counting of colonies was performed with an automatic colony counter Scan 4000 Ultra HD (Interscience, Saint Nom la Bretêche, France). Finally, the results were expressed as the logarithm of the count of Colony Forming Units in one gram of bedding material (log CFU/g).

### 2.3. Testing the Bedding Materials in Operational Conditions Involving Animals—Experiment Design and Sample Collection

**Figure 1:**
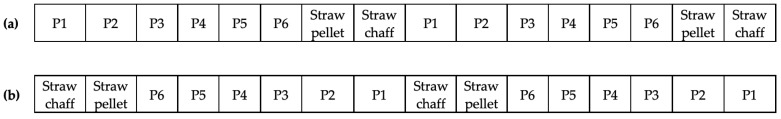
Experimental setting—allocation of bedding materials to compartments with animals: (**a**) 1st experimental cycle; (**b**) 2nd experimental cycle.

The experiment was performed in two 42-day-long cycles at the Agricultural Experimental Station Wilanów Obory (Warsaw University of Life Sciences, Warsaw, Poland). The first experimental cycle took place from 4 May to 6 July 2020, and the second one from 31 August to 12 October 2020. On the first day of each cycle, the eight tested materials (P1–P6, 100% straw pellets, and straw chaff) were put as beddings into the compartments, ensuring 1.1 kg of each material per 1 m^2^. Each material was placed in two compartments for replication (Figure 1).

**Figure 2:**
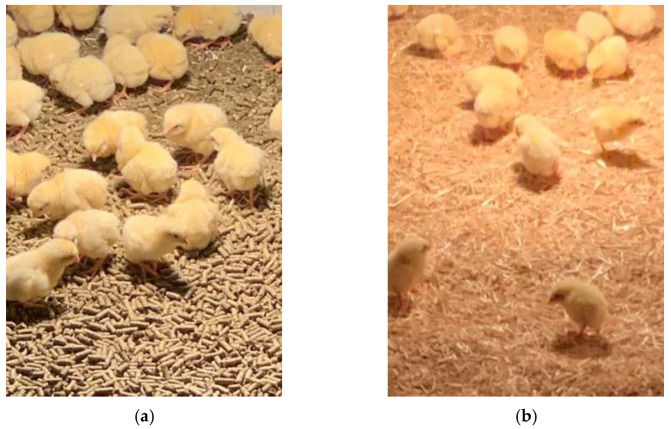
Experimental setting—1st day of bedding materials use by animals: (**a**) pelletised materials containing parts of medicinal plants; (**b**) straw chaff (control).

Then, 75 Ross 308 broiler chickens were reared in each compartment at a mean rate of 11.4 birds per 1 m^2^ (Figure 2). The animals were kept in standard farm conditions, in line with the recommendations of the producer of genetic material (Aviagen, Obrowo, Poland), as well as following the relevant national regulations setting requirements for keeping of farm animals [37].

The study was performed without causing any harm to the health and welfare of the animals participating in the experiment or affecting their routines. Since the study did not involve any actions that would not be performed during standard farming practices, the research implementation did not require ethical approval under the relevant national regulation on the protection of animals used for scientific purposes [38].

A sterile collection of mixed bedding samples, each weighing 200 g ± 10 g, was taken every 7 days from each compartment. On the same day, the samples were transported under refrigerated conditions for analysis to the Natural Products Laboratory (Research and Innovation Centre Pro-Akademia, Konstantynów Łódzki, Poland). Three independent samples were prepared for each analysis (three repetitions). The first repetition was the sample of the specific bedding material taken from one compartment. The second repetition was the sample taken from the other compartment with the same material. The third repetition was the sample taken in ratio 1:1 from both compartments.

### 2.4. Physico-Chemical and Microbiological Characterisation of Materials during Their Exploitation

#### 2.4.1. Moisture Content

The mean water content in the bedding material was determined according to the oven method (105 °C) [36].

#### 2.4.2. pH Value

The pH value was measured with a pH meter Lab 850 (SiAnalytics GmbH, Mainz, Germany). A pH meter probe was inserted into 25 g of the material suspended in 300 mL of Maximum Recovery Diluent (Oxoid, Hampshire, UK) and the pH value was read.

#### 2.4.3. Total Count of Aerobic Mesophilic Bacteria, the Total Count of Yeasts and Moulds

Samples of 25 g of each bedding were weighed in sterile filter bags (BagFilter P, Interscience, Saint Nom la Bretêche, France) containing 300 mL of Maximum Recovery Diluent (Oxoid, Hampshire, UK) and ground in a stomacher homogeniser (Bag Mixer 400 LW, Interscience, Saint Nom la Bretêche, France). Series of ten-fold dilutions were made from the suspension of bedding after grounding and inoculated directly on the surface of the appropriate selective medium by an automatic spiral plater (easySpiral^®^Pro, Interscience, Saint Nom la Bretêche, France). Each sample dilution (0.05 mL) was surface inoculated on tryptone soya agar (Oxoid, Hampshire, UK) for a total count of aerobic mesophilic bacteria, Sabouradud’s medium with chloramphenicol (Oxoid, Hampshire, UK) for a total count of yeasts and moulds and incubated at 30 °C for 48–72 h in an air-jacketed incubator (NuAire 5831, NuAire Inc., Plymouth, MN, USA). Counting of colonies was performed with an automatic colony counter Scan 4000 Ultra HD (Interscience, Saint Nom la Bretêche, France). Finally, the results were expressed as the logarithm of the count of Colony Forming Units in one gram of bedding material (log CFU/g).

#### 2.4.4. Total Count of *Staphylococcus* sp., *Escherichia* sp., *Listeria* sp., *Salmonella* sp., and *Candida* sp.

For this, 0.05 mL of each sample dilution was plated on selective chromogenic media: CHROMagar™ *Salmonella* PLUS for *Salmonella* sp., *Staphylococcus* CHROMagar™ for *Staphylococcus* sp., CHROMagar™ ECC for *Escherichia* sp., CHROMagar *Listeria*™ for *Listeria* sp., and CHROMagar™ media *Candida* for *Candida* sp. (CHROMagar Microbiology, Paris, France). The plates were incubated in an air-jacketed incubator (NuAire 5831, NuAire Inc., Plymouth, MN, USA) at 37 °C for the following periods: 24 h for *Salmonella* sp., *Staphylococcus* sp., and *Escherichia* sp., and 48–72 h for *Listeria* sp. and *Candida* sp. Counts of each microorganism group were calculated based on the characteristic growth described in the manufacturer’s medium manual [39]. Counting of colonies was performed with an automatic colony counter Scan 4000 Ultra HD (Interscience, Saint Nom la Bretêche, France). Finally, the results were expressed as the logarithm of the count of Colony Forming Units in one gram of bedding material (log CFU/g).

### 2.5. Statistical Analysis

The statistical analysis of the results was performed using the STATISTICA 13 (Dell, Round Rock, TX, USA). To investigate variability between different samples, data were subjected to one-way analysis of variance (ANOVA), followed by the Tukey honest significance test (HSD) at the significance level α = 0.05.

## 3. Results

### 3.1. Physicochemical and Microbiological Parameters of Materials before Exploitation

The essential oil content in the tested bedding materials ranged between 0.09 and 0.35 mL/100 g (Table 2). The least essential oil content was found in materials P1 and P6, which resulted from high *Origanum vulgare* (oregano) herb content in the recipe. Materials P2, P3, and P4 were characterised by the highest essential oil content. The water absorption ranged between 242.43% and 392.27%. The level of pH in bedding materials ranged between 6.20 and 6.55. The initial total count for aerobic mesophilic bacteria ranged from 5.00 to 7.20 log CFU/g.

### 3.2. Physico-Chemical Characterisation of Materials during Their Exploitation

#### 3.2.1. Moisture Content

**Figure 3:**
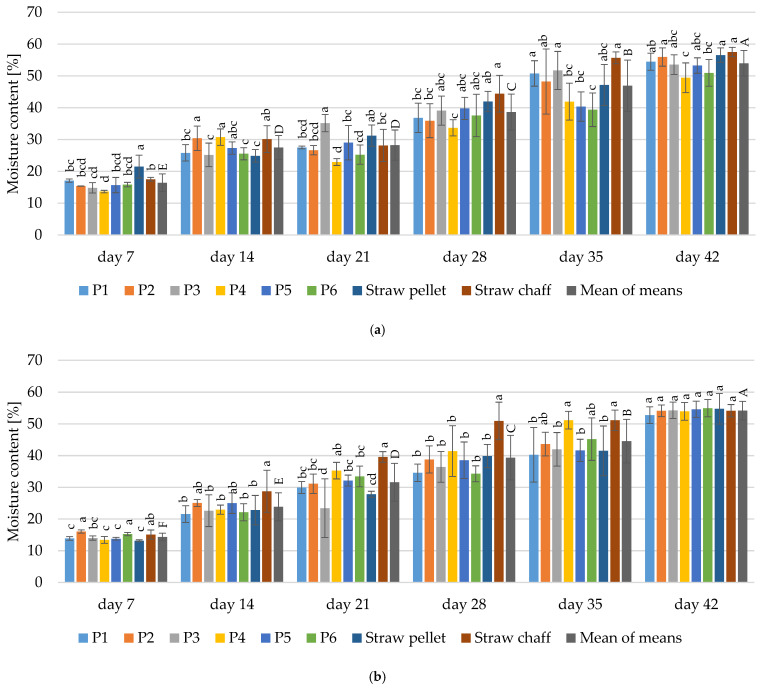
Changes in the moisture content in the tested bedding materials during: (**a**) 1st experimental cycle; (**b**) 2nd experimental cycle. All values are means of three independent replicates ± SD, except the last bars for each day, which are the means-of-means. For each day, means with a common lowercase letter are not significantly different by the Tukey test at the 5% level of significance. For different days, means-of-means with a common uppercase letter are not significantly different by the Tukey test at the 5% level of significance. Pairwise comparisons between materials are presented only for observations from the same day. Pairwise comparisons between days are presented only for means-of-means. No pairwise comparisons between cycles are presented.

The moisture content determined in the bedding materials after 7 days of their use was relatively low in both experimental cycles and ranged between 13.66% and 21.49% (Figure 3). The moisture content in the materials increased with the duration of each experimental cycle and was, on average, 27.51% (on day 14), 28.25% (on day 21), 38.66% (on day 28), 46.91% (on day 35), and 53.97% (on day 42) in the first cycle. In the second cycle, the average moisture content determined in the bedding materials was 23.86% (on day 14), 31.58% (on day 21), 39.35% (on day 28), 44.56% (on day 35) and 54.19% (on day 42).

#### 3.2.2. pH Value

The mean pH value of the tested bedding materials, measured during the first experimental cycle was 6.41 on days 7 and 14 (Table 3). On day 7, a significantly lower pH value than in the straw pellets was observed in the material P1 (6.33 vs. 6.48). After 14 days, the bedding materials P2 and P4 had a significantly lower pH value compared to the straw pellets (6.30 vs. 6.65, respectively). On the other dates, no significant differences were found in the pH value between the samples within the term. The average pH on day 21 was 7.0; on day 28—7.3; on day 35—7.3, and on day 42—6.9. The pH value of the tested materials, measured during the use in the second cycle, was on average 6.35 on days 7 and 14 (Table 4). On days 21, 28, and 35 there were significant differences between the pH value of the samples within the term. On day 14, samples of the prototype bedding materials P1, P3 and P5 were characterised by a significantly lower pH value to straw chaff. The pH value for the litter on day 21 was 6.65; on day 28—7.54; on day 35—7.34; and 7.44 on 42 day of use.

### 3.3. Total Count of Aerobic Mesophilic Bacteria, the Total Count of Yeasts and Moulds in Materials during Use under Operating Conditions

The contamination with aerobic mesophilic bacteria depended on the duration of material exploitation in both experimental cycles (*p* ≤ 0.05) (Table 5 and Table 6). It also depended on the bedding material on days 7 and 14 of the first experimental cycle and days 7, 14, 21, 35 and 42 of the second cycle (*p* ≤ 0.05). We found a significant increase in contamination with mesophilic aerobic bacteria along with the time of bedding use (*p* ≤ 0.05) during both experimental cycles. The bacterial mean count after 42 days of the experiment was, on average, 6 logs higher in the first cycle, and over 3 logs higher in the second cycle than at the beginning of the experiment.

In the first experimental cycle, the mean log count of aerobic bacteria in materials was 8.98 log after 7 days, and 9.53 log CFU/g after 14 days. After 21 days of use, the count of mesophilic aerobic bacteria in the bedding materials increased by 1–2 logs, compared to day 14, and averaged 11.25 log CFU/g. After 28 days, the count of mesophilic aerobic bacteria determined for all the tested samples was above 15 logs CFU/g and remained at this level until the end of the experiment (day 42). For the P1, P2, and P4 bedding materials, after 7 days of experiments we observed a smaller number of the total count of microorganisms—1 log less in comparison to straw pellets (8.22, 8.49 and 8.64 vs. 9.63 log CFU/g, respectively). Additionally, after 14 days, significantly less mesophilic aerobic bacteria in comparison to straw pellets were found in P1, P3, P4, and P5 bedding materials. In subsequent dates, we observed no significant differences in the count of mesophilic bacteria between the tested materials.

In the second experimental cycle, the total count of aerobic bacteria in the tested materials after 14 days of use ranged between 9.47 and 10.46 log CFU/g. We found significantly more bacteria in straw chaff than in material P1 on day 7, and significantly more bacteria in the straw chaff than P1, P3, P5, and P6 on day 14. After 28 days, the count of mesophilic aerobic bacteria in the materials did not differ significantly between the samples, and was, on average, 11.22 log CFU/g. After 35 days, we observed a reduction in the total count of microorganisms above 1 log in material P3 when compared to the straw pellets (11.86 vs. 13.02 log CFU/g). A reduction of the total count of microorganisms in comparison to the straw pellets also occurred after 42 days of the experiment for materials P1–P3 (12.75; 12.92; 12.78 vs. 14.12 log CFU/g, respectively).

The contamination with yeast and moulds depended on the duration of material exploitation in both experimental cycles (*p* ≤ 0.05) (Table 7 and Table 8). It differed between materials on days 7, 21, 28, and 42 during the first experimental cycle, and on days 7, 14, 21, and 42 during the second cycle. In both cycles, we observed an increase in contamination with yeasts and moulds in all bedding materials until 21 days of the experiment (*p* ≤ 0.05). After the inflection date, we observed a gradual decrease in this group of microorganisms. This was related to the parallel increase in the count of bacteria in the materials and the increasing pH, which were unfavourable for the development of microbes. After 7 days, the total count of yeast and moulds ranged between 5.3 log CFU/g and 7.08 CFU/g in the first cycle, and between 6.40 CFU/g and 7.04 CFU/g in the second cycle. Contamination with yeasts and moulds after 7 days of the first cycle was lower than in the straw pellet and chaff for the P4 material only. At the end of the second experimental cycle, contamination with yeasts and moulds was lower for P5 and P6 bedding materials in comparison to the straw pellets (5.45 and 5.48 log CFU/g vs. 6.21 log CFU/g).

### 3.4. Count of Microorganisms in Bedding Materials during Use under Operating Conditions

#### 3.4.1. *Staphylococcus* sp.

The contamination with *Staphylococcus* sp. depended on the duration of material exploitation in both experimental cycles (*p* ≤ 0.05). The contamination depended on the bedding material on days 7, 14 and 21 in the first cycle, and days 7, 14 and 35 for the second cycle (*p* ≤ 0.05).

**Figure 4:**
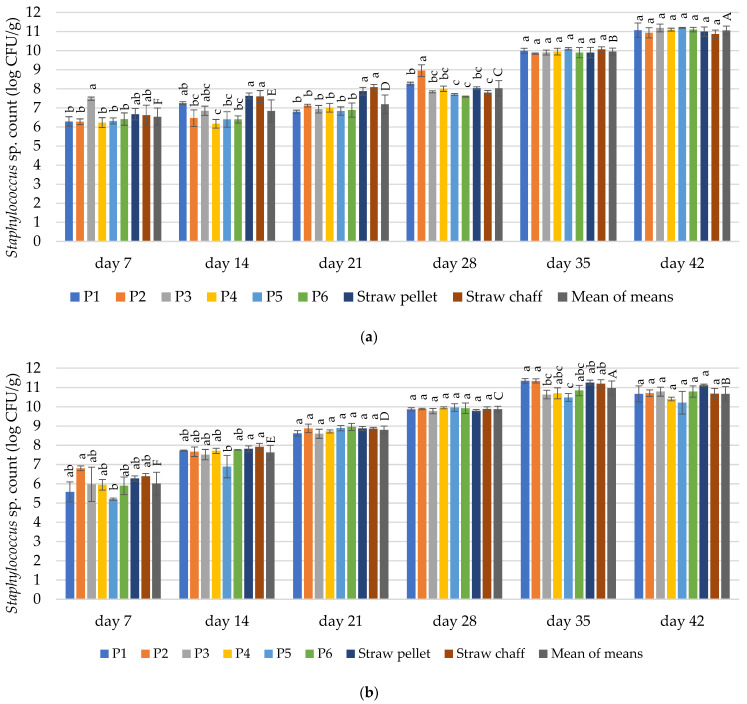
Changes in the count of microorganisms of *Staphylococcus* sp. in the tested bedding materials: (**a**) 1st experimental cycle; (**b**) 2nd experimental cycle. All values are means of three independent replicates ± SD, except the last bars for each day, which are the means-of-means. For each day, means with a common lowercase letter are not significantly different by the Tukey test at the 5% level of significance. For different days, means-of-means with a common uppercase letter are not significantly different by the Tukey test at the 5% level of significance. Pairwise comparisons between materials are presented only for observations from the same day. Pairwise comparisons between days are presented only for means-of-means. No pairwise comparisons between cycles are presented.

In the first experimental cycle, the count of *Staphylococcus* sp. in all tested materials after 7 days ranged between 6.27 and 7.48 log CFU/g (Figure 4a). After 14 days, we detected significantly fewer bacteria (<6.46 log CFU/g) in P2, P4, P5, and P6 materials compared to straw pellets and chaff (>7 logs CFU/g). We also observed this effect after 21 days of use for P1–P6 bedding materials. After 28 days, significantly more *Staphylococcus* sp. bacteria was found for materials P1 and P2 in comparison to P5, P6 and straw chaff. On days 35 and 42, we observed a high contamination with *Staphylococcus* sp. in all samples (9.95 log and 11.05 log CFU/g, respectively).

In the second experimental cycle, we observed an increase in the count of *Staphylococcus* sp. until 35 days of use (Figure 4b). The count of *Staphylococcus* sp. detected in bedding materials after 7 days of use in the second cycle ranged between 5.58 and 6.38 log CFU/g. On day 14, we found significantly less *Staphylococcus* sp. in the bedding material P5 as compared to the straw pellet and chaff (6.89 vs. 7.81 and 7.92 logs CFU/g, respectively). After 21 and 28 days, we found no differences in contamination between all tested materials. Similarly to the 14 day, on day 35, the count of *Staphylococcus* sp. was significantly lower for the material P5 in comparison to the straw pellets (10.48 vs. 11.25 log CFU/g). After 42 days, we found no significant differences in *Staphylococcus* sp. mean count between the materials (10.67 log CFU/g).

#### 3.4.2. *Escherichia* sp.

**Figure 5:**
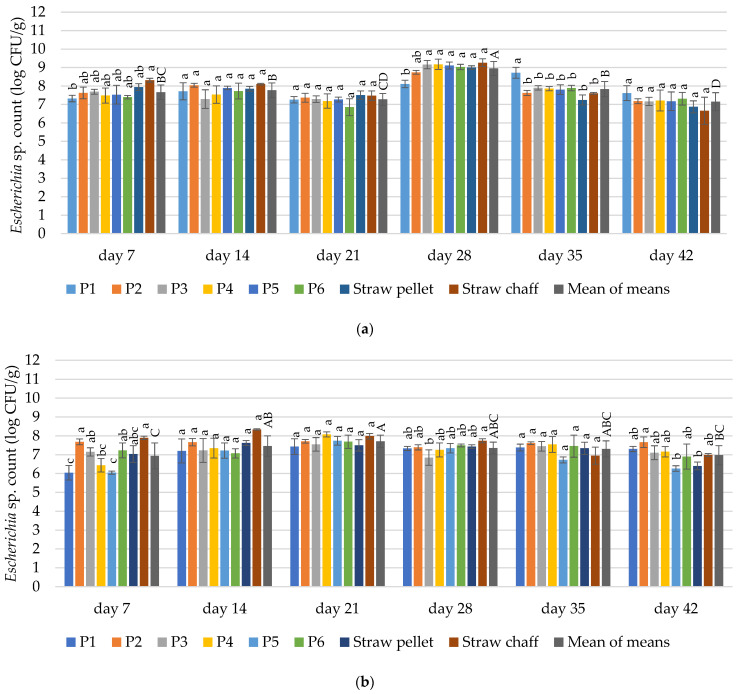
Changes in the count of microorganisms of *Escherichia* sp. in the tested bedding materials: (**a**) 1st experimental cycle; (**b**) 2nd experimental cycle. All values are means of three independent replicates ± SD, except the last bars for each day, which are the means-of-means. For each day, means with a common lowercase letter are not significantly different by the Tukey test at the 5% level of significance. For different days, means-of-means with a common uppercase letter are not significantly different by the Tukey test at the 5% level of significance. Pairwise comparisons between materials are presented only for observations from the same day. Pairwise comparisons between days are presented only for means-of-means. No pairwise comparisons between cycles are presented.

The contamination with *Escherichia* sp. was dependent on the duration of material exploitation in both cycles (*p* ≤ 0.05). The contamination was dependent on the bedding material on day 7, 28 and 35 in the first cycle, and on day 7, 28 and 42 for the second cycle (*p* ≤ 0.05) (Figure 5).

Contamination of the bedding materials with *Escherichia* sp. ranged from 7.32 to 8.31 log CFU/g in the first two terms of the first cycle. Only the P1 bedding material was less contaminated with *Escherichia* sp. in comparison to the straw chaff on day 7. No significant differences in the *Escherichia* sp. count were found between tested samples after 14 days and 21 days. After 28 days, a significant increase in the count of *Escherichia* sp. was noted for all bedding materials (up to 9.26 log CFU/g). The mean reduction in the number of mentioned microbes on days 35 and 42 was observed (mean contamination 7.83 and 7.15 log CFU/g). The exception was the prototype bedding material P1 where, after 35 days, the contamination was higher than in other bedding materials (8.71 log CFU/g). On day 42 of use, no significant differences were found in the contamination of *Escherichia* sp. between tested samples.

In the second cycle, after 7 days of the experiment, the contamination of bedding materials with *Escherichia* sp. was 6.94 log CFU/g on average. A significantly lower count was found for the prototype bedding material P1 and P3 compared to straw chaff (6.04 vs. 7.89 log CFU/g). After 14, 21, and 35 days of use, no significant differences were found in the contamination with *Escherichia* sp. between samples. On day 14 the average *Escherichia* sp. count was 7.46; on day 21—7.7; on day 28—7.35; on day 35—7.30 log CFU/g. No significant differences in contamination were found between bedding materials P1–P6 and straw chaff after 42 days. More *Escherichia* sp. (7.66 log CFU/g) was determined in the P2 bedding material compared to the straw pellets and material P5 within this term.

#### 3.4.3. *Listeria* sp.

**Figure 6:**
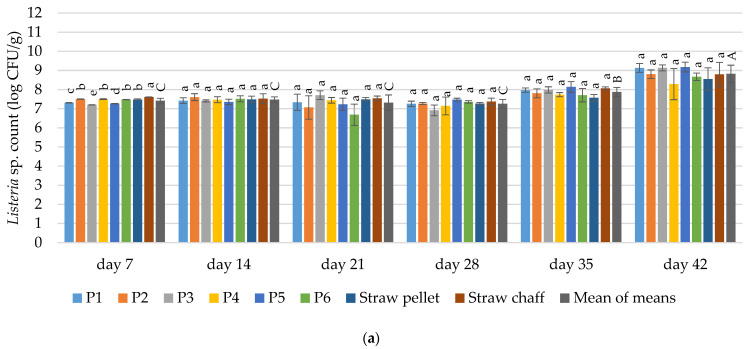

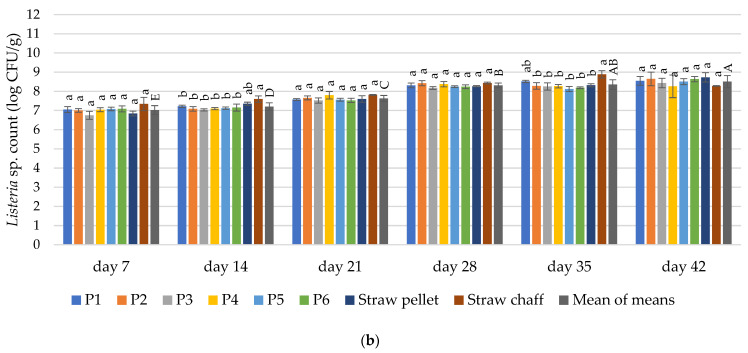
Changes in the count of microorganisms of *Listeria* sp. in the tested bedding materials: (**a**) 1st experimental cycle; (**b**) 2nd experimental cycle. All values are means of three independent replicates ± SD, except the last bars for each day, which are the means-of-means. For each day, means with a common lowercase letter are not significantly different by the Tukey test at the 5% level of significance. For different days, means-of-means with a common uppercase letter are not significantly different by the Tukey test at the 5% level of significance. Pairwise comparisons between materials are presented only for observations from the same day. Pairwise comparisons between days are presented only for means-of-means. No pairwise comparisons between cycles are presented.

The contamination with *Listeria* sp. was dependent on the duration of the materials’ exploitation in both cycles (*p* ≤ 0.05). The contamination with *Listeria* sp. did not depend on the bedding material in the first cycle from 14 days of use until the end of the experiment. It differed between bedding materials on day 14 and 35 in the second cycle (*p* ≤ 0.05) (Figure 6).

In the first experimental cycle, the count of *Listeria* sp. after three first examined terms ranged from 6.69 to 7.61 log CFU/g. Significantly more of *Listeria* sp. was detected in the straw chaff in comparison to other bedding materials on day 7. An increase in the contamination with *Listeria* sp. was observed at the end of the experiment (day 35 and 42). Above 8 log CFU/g was determined in all tested bedding materials on day 42 (see Figure 4a).

Significantly less *Listeria* sp. was found after 14 days of use in all prototype bedding materials (P1–P6) than in straw chaff during the second cycle. The increase in contamination in the second cycle was more dynamic than in the first cycle. The average *Listeria* sp. count was increasing from 28 until 42 day of the experiment (See Figure 4b).

#### 3.4.4. *Salmonella* sp.

The contamination with *Salmonella* sp. was dependent on the duration of the experiment in both experimental cycles (*p* ≤ 0.05). The contamination with *Salmonella* sp. was dependent on the bedding material in almost all terms in the first cycle (42 days was the exception), and on days 7, 14, 21, 35 and 42 for the second cycle (*p* ≤ 0.05).

**Figure 7:**
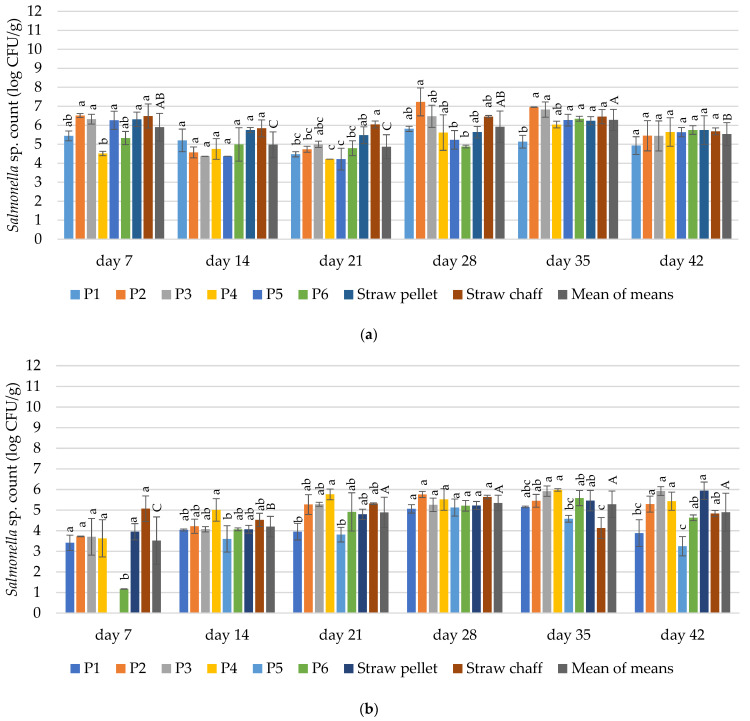
Changes in the count of microorganisms of *Salmonella* sp. in the tested bedding materials: (**a**) 1st experimental cycle; (**b**) 2nd experimental cycle. All values are means of three independent replicates ± SD, except the last bars for each day, which are the means-of-means. For each day, means with a common lowercase letter are not significantly different by the Tukey test at the 5% level of significance. For different days, means-of-means with a common uppercase letter are not significantly different by the Tukey test at the 5% level of significance. Pairwise comparisons between materials are presented only for observations from the same day. Pairwise comparisons between days are presented only for means-of-means. No pairwise comparisons between cycles are presented.

After 7 days of the first cycle, the count of *Salmonella* sp. was significantly lower for the P4 bedding material (4.51 log CFU/g) when compared to the straw pellets and chaff (6.31 and 6.49 log CFU/g, respectively). On day 14, the average count for *Salmonella* sp. for the tested materials was 4.98 log CFU/g. On day 21, significantly lower contamination with *Salmonella* sp. was determined in the P1, P2, P4, P5 and P6 bedding materials compared to the straw chaff. The P4 and P5 bedding materials were less contaminated than straw pellets within this term (4.21 and 4.22 vs. 5.48 log CFU/g). On day 28, an average count for *Salmonella* sp. was 5.92 log CFU/g. On day 35, significantly less *Salmonella* sp. was found in the P1 bedding material than in straw pellets and chaff (5.14 vs. 6.24 log and 6.46 CFU/g, respectively). After 42 days of use, no significant differences in *Salmonella* sp. contamination between the samples were observed (Figure 7a).

After 7 days of bedding use in the second cycle, no *Salmonella* sp. was found in the P5 prototype. Significantly lower contamination in comparison to straw pellets and chaff was observed for the P6 bedding material (1,17 vs. 3.95 and 5.07 log CFU/g) within the term. There were no differences in contamination between straw pellets and chaff, and the P1, P2, P3 and P4 bedding materials. Relatively low amounts of *Salmonella* sp. were found on day 21 in P1 and P5—3.95 and 3.8 log CFU/g. On day 28, no significant differences were found between the samples. Significantly less *Salmonella* sp. was determined in the P5 bedding material in comparison to the straw pellets (3.88 and 3.24 vs. 5.94 log CFU/g) on day 42 (Figure 7b).

#### 3.4.5. *Candida* sp.

**Figure 8:**
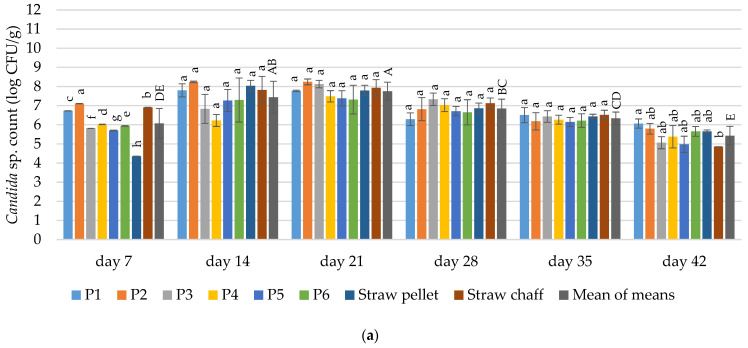

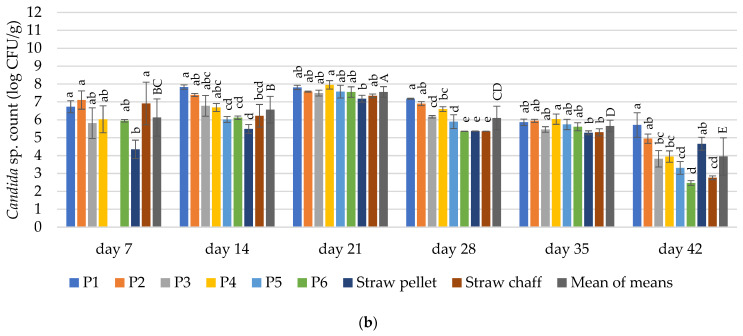
Changes in the count of microorganisms of *Candida* sp. in the tested bedding materials: (**a**) 1st experimental cycle; (**b**) 2nd experimental cycle. All values are means of three independent replicates ± SD, except the last bars for each day, which are the means-of-means. For each day, means with a common lowercase letter are not significantly different by the Tukey test at the 5% level of significance. For different days, means-of-means with a common uppercase letter are not significantly different by the Tukey test at the 5% level of significance. Pairwise comparisons between materials are presented only for observations from the same day. Pairwise comparisons between days are presented only for means-of-means. No pairwise comparisons between cycles are presented.

The count of *Candida* sp. depended on the duration of the experiment and bedding materials in both experimental cycles (*p* ≤ 0.05) (Figure 8). In the first cycle, after 14 days of the experiment, the average number of *Candida* sp. was 7.44; after 21 days was 7.75; after 28 days was 6.85; and was 6.33 log CFU/g after 35 days. The differences between the bedding materials in the first cycle were noted only on day 7 and 42. After 7 days, the lowest number of *Candida* sp. was found in the straw pellets. The highest contamination was found for straw chaff. On day 42, the count of *Candida* sp. was significantly higher in the P1 bedding material in comparison to the straw chaff (6.06 vs. 4.86 log CFU/g). Along with the decrease in the number of yeasts after the 21 days of use, an increase in the total number of bacteria in the bedding materials was observed (Table 5 and Table 6), which suggests a strong competition between microorganisms from the group of bacteria and yeasts. Better conditions for the growth of bacteria in the beddings are confirmed by the pH values determined for litter during use (Table 3 and Table 4).

In the second cycle, after 7 days of use, no *Candida* sp. was found in the prototype bedding material P5. The log mean for the number of *Candida* yeasts was 6.12 CFU/g, and the lowest number of *Candida* sp. was found in the straw pellets. The lowest contamination on day 28 was found for the P6 bedding material, straw pellets and straw chaff.

Until the 21st day of use, contamination of the bedding materials with *Candida* sp. was increasing. After 14 days of the experiment, the average count for *Candida* sp. was 6.57; after 21 days was 7.56; and was 6.1 log CFU/g after 28 days. On days 35 and 42, the mean count for *Candida* sp. was 5.65 and 3.65 log CFU/g, respectively. On day 35, significantly more *Candida* sp. was found in the bedding material P4 in comparison to the straw pellets. On day 42, significantly less *Candida* sp. was found in the bedding material P6 and straw chaff in comparison to straw pellets (2.46 and 2.76 vs. 4.65 log CFU/g).

## 4. Discussion

### 4.1. Physicochemical Parameters of Bedding Materials Prior and during Their Exploitation

#### 4.1.1. Water Absorption Capacity and Moisture Content

Water absorption capacity is the most important quality parameter of bedding materials [40]. Wet beddings have been associated with contact dermatitis and other flock infections in poultry, as well as lowered feed conversion ratios [41]. In this context, our results go beyond previous reports on the water absorption capacity of bedding materials. The water absorption capacity in most of the tested materials (P1, P3–P6) ranged between 346.43 and 392.27%, making them superior not only to our controls, but also to other bedding materials, such as flax shive and wood shavings (292% and 181.25%, respectively) [42]. Our results are consistent with previous studies that reported the water absorption capacity for wheat and barley fibers ranging from 270 to 400% [43]. Both studies mentioned have followed similar experimental conditions for water absorption capacity assessment to ours (soaking for 30 min in room temperature).

When analysing our results against those of other prior studies on animal bedding materials [44,45,46,47,48], it must be pointed out that comparisons should be performed with caution, since there are several methods of measuring this parameter. In particular, the duration of samples soaking, which in prior studies ranged between 2 min [46] and 24 h [47], influences the water absorption rates [44]. Furthermore, it can be also affected by the water removal approach, e.g., involving the application of a vacuum [48] or centrifuging [40].

With regard to the moisture content, good practice recommends bedding materials that have a moisture content of less than 10%, which was slightly higher in our study. During the poultry production cycle the moisture should be maintained between 20% and 25%, but it may reach 35% [49]. These parameters were maintained by the bedding materials until the 14th day of use for all tested bedding materials containing parts of medicinal plants and straw pellets.

#### 4.1.2. pH

In all studied materials we observed relatively low and similar pre-exploitation pH levels, which were in the lower part of the pH range regarded as optimal for most bacteria growth (pH 6–8) [50]. With regard to the materials exploitation phase, our results do not confirm a significant effect of the use of the tested materials containing parts of medicinal plants on the pH levels as compared to the control (straw pellets and straw chaff). However, after 42 days of exploitation, the pH levels in all tested materials were lower than in earlier studies on rice hulls (pH 8.13–8.97) [15,51]. Similar results to ours were reported previously for rye straw used as poultry bedding materials over 6 weeks (pH 7.3) [32].

#### 4.1.3. Essential Oil Content

Due to the documented activity of essential oils against microbial threats in poultry production [20,21,22,23,24], they have been studied in two poultry production applications: animal feed supplementation [52,53,54] and antimicrobial fogging of poultry houses [55]. Our study expands the literature on applications of essential oils as active components of poultry bedding materials. Furthermore, our results are among the few reports on the changes in essential oil content during production of materials containing such substances. The evaporation of essential oils from biobased materials can be forced due to an increased temperature, e.g., during the drying of leaves of French tarragon (*Artemisia dracunculus*) at 60 °C a loss of 75% of the essential oil content was reported [56]. It can also occur spontaneously over storage time, e.g., a 43% drop in essential oil content in pelletised hops was reported over 30 days [57]. The observed difference between the essential oil content in the input ingredients before processing and in the produced materials (following processing in an increased temperature and pressure) varied between 51% and 67%, depending on the material. This is consistent with observations of the aforementioned prior studies on the impacts of drying and storage on the essential oil content in other biobased materials.

Plants used in our study are industrially important medicinal crops, widely cultivated and distributed in many parts of the world, also in Poland, where the investigated materials were produced. They are rich in essential oils. Savory (*Satureja hortensis*) and oregano (*Origanum vulgare*) herbs are the main components of all tested bedding materials. Their essential oils have widely documented antimicrobial properties in poultry production [20,21]. Therefore, the potent antimicrobial activity of the tested bedding materials (P1–P6) is due to the high content of these two ingredients. *Mellissa officinalis*, *Salvia officinalis* and *Thymus vulgaris* stems contain smaller, yet noticeable amounts of essential oils, whose activities against microbials present in poultry production have also been demonstrated [22,23,24]. These raw materials are typically treated as wastes of herbal production. The purpose of their use in the studied materials was not only to improve the expected antimicrobial properties, but also the economics of materials production.

The presented results could be affected by two important properties of essential oils of medicinal plants: the essential oil content and its composition. Both characteristics are likely to influence the antimicrobial activity of materials comprising parts of medicinal plants. The essential oil content and composition in raw materials acquired from medicinal plants depend on many factors, which can be divided into primary and secondary ones. The primary factors include: (1) plant species [58], (2) plant organs and the development phase [59], (3) intraspecific genetic variability of plants [60] and (4) their growing conditions, e.g., exposure to biotic and abiotic stress [61], and soil fertility [62]. The secondary factors concern conditions of post-harvest raw materials’ processing, e.g., temperature and time of raw material storage [63] or heat treatment. The negative effect of heat treatment is the loss of essential oil content, which in our research reached 61%. However, the heat treatment may also entail positive effects. Prior studies have demonstrated that the high temperature (≥100 °C) caused the conversion of the essential oil components from savory (*Satureja hortensis*) [64]. It was observed that the concentration of carvacrol, which is one of the most potent antimicrobial constituents of *S. hortensis* essential oil, increased after heat treatment. Therefore, the pelletisation could have led to a higher antimicrobial activity of the materials in the present study as compared to the antimicrobial activity of separate, raw ingredients, even despite the evaporation of essential oils during the materials production. Further research is needed to investigate the trade-off between the counteracting effects of pelletisation on the antimicrobial activity of materials.

### 4.2. Total Count of Aerobic Mesophilic Bacteria in Materials Prior to Their Exploitation

According to prior studies, the total count of mesophilic aerobic bacteria in bedding materials before use can vary between 4–7.44 log CFU/g [26]. We reported a mean contamination of bedding materials prior to their exploitation at the level of 5.62 log CFU/g, which is consistent with the aforementioned earlier reports. Only the material P5 was characterised by a relatively high total count of mesophilic aerobic bacteria (mean count 7.2 log CFU/g). This is likely to be caused by the fact that these samples were the only one to contain thyme (*Thymus vulgaris*) stems, which could have been more contaminated than other raw herbal materials used for materials production.

It should be noted that the microbiological quality of the bedding material before its use depends to a large extent on the quality of the materials it contains, as well as the technology of its production. With regard to dry medicinal plants, the total microbial count may vary within a range in 2–4 log CFU/g for thyme, 2–6 log CFU/g for oregano, and other herbal dries [65]. The pelletisation process, which involves high pressure and temperature, promotes the sanitisation of those ingredients [66].

### 4.3. Diversity and Dynamics of Microorganisms over the Exploitation Period

#### 4.3.1. Total Count of Aerobic Bacteria and Total Count of Yeasts and Moulds in Materials

Studies performed by other authors show at least 1 log increase in CFU/g for a total count of aerobic bacteria in bedding material used in poultry houses with broilers during the first 3 weeks of exploitation [26,32]. The increasing trend for total aerobic bacteria is in line with our results. The average contamination of examined materials during the mentioned period in our study was 9.73 log CFU/g. As it was previously shown, further exploitation of poultry bedding material results in the further development of total aerobic microbial flora to 8–10.5 log CFU/g [26,27,28,29,30,31,32]. We observed a mean total count of 12–16 log CFU/g in the period of 4–6 weeks of exploitation. These high values may be caused by the different densities of broilers per square metre in the house, as well as moisture content, which is responsible, to a great extent, for microbial diversity in litter [28]. Future studies should report more details on these aspects, as they are important for drawing conclusions.

What is more, it should be noted that in our research, we observed significant differences in the proportions of microorganisms in individual materials. The effect of the external application of plant-derived substances such as thyme and peppermint oil (fogging) on total aerobic bacteria microflora was observed in a previous study [55]. The reduction of the total microbial count was observed after the external application of quicklime onto wood shavings and rice hulls [67].

To the best of our knowledge, this study is the first where the interaction between materials containing parts of medicinal plants and poultry microflora was examined. Differences with total aerobic microflora in relation to straw pellets and chaff within the first 14 days of use were found in material P1, with high oregano content (24%), supplemented with savory (6%). The dominant component of the essential oil of oregano is carvacrol (over 70%), followed by γ-terpinene and p-cymene [68,69]. Carvacrol and thymol were reported as the main constituents of savory essential oil (over 20% of total essential oil each) [70]. Antimicrobial activity of oregano essential oils was reported against gram-positive pathogens (*Bacillus cereus* and *B. Subtilis*) and some gram-negative bacteria (*E. coli* but not *Salmonella* species). It was reported that high carvacrol and thymol (which is phenolic) are responsible for the antimicrobial activity of essential oils [71].

Correspondingly to aerobic bacteria, contamination with yeasts and moulds during the use of a bedding material can be related by its content. It was found that rye straw, wheat straw, and mixed straw chaff were characterised by the high number of moulds, ranging between 15.3 and 28.4 log CFU/g, whereas in sawdust it was significantly less contaminated (7.2 log CFU/g). The best results were achieved for wood chips (3.1 log CFU/g). The authors suggest that the lower fungal content may be related to the presence of antimicrobial compounds in wood as shown by some of our research results (see *Candida* sp., day 35, 1st cycle) [27]. Even though we did not replicate these previous reports (no significant differences in yeast and mould contaminations were found in the materials we tested), our results suggest a strong competition between microorganisms: bacteria vs. yeasts and moulds.

An important question concerns the plants used in the study. If the fragmented plants are used, their biologically active components must be released to the outside. It should be assumed that it is caused by high humidity induced by bird excreta, (>50% of water, 20% of organic matter) which causes maceration, and bioactive substances from plants are released into the environment where they can interact with microbes. Essential oil components are hydrophobic and cannot be fully extracted from plant material and, therefore, their performance in the litter may be limited. Carvacrol due to its hydrophobicity can be accumulated in the microbial cell membrane and may induce conformational modification of the membrane resulting in the microbial cell death [72]. The antimicrobial effect of plant extracts may be forced by heat treatment. It was found that poultry litter compost subjected to a physical heat treatment reduced pathogenic bacteria, such as *Salmonella enterica* and *Enterococcus faecium* [73]. Plant components combined with heat treatment may reduce significantly the number of pathogenic microbes in poultry litter. However, further study must be performed because sanitisation of the used litter (bird excreta and bedding material) was not the subject of our study. It is promising that in vitro studies showed that essential oil compounds present in savory and oregano (carvacrol and thymol) combined with moderate heat (55 °C) significantly reduced the population of *Listeria monocytogenes* [74].

#### 4.3.2. *Staphylococcus* sp.

*Staphylococci* can account for approximately 90% of all culturable bacteria in poultry bedding materials during their exploitation (observation based on data concerning an unspecified material type) [75]. *Staphylococcus* sp. widely occurs in the litter and comes from the birds excreta [35]. Therefore, its significantly lower presence observed in the P5 material after 14 days of bedding exploitation in both experimental cycles is an important finding. This may be caused by the mass share of *Thymus vulgaris* stems in combination with the *Satureja hortensis* herb in this material among all tested samples, which are both rich in thymol [70,76]. The essential oil contained in thyme applied in low concentration has proven a significant potential for inhibiting *Staphylococci* growth [77]. The present study confirmed the findings about the suitability of using thyme as a factor limiting the growth of *Staphylococcus* sp. bacteria. We observed a 0.5–1 log reduction of *Staphylococcus* sp. on bedding material P5 in relation to straw pellets and chaff. A similar effect was observed after the application of thyme oil on straw chaff during material exploitation [55], while a stronger effect was reported after the application of quicklime (CaO) on rice hulls and wood shavings [67]. However, it must be pointed out that the application of aggressive chemical agents could potentially result in burning the soles of animals.

#### 4.3.3. *Escherichia* sp.

Since *Escherichia* sp. develops in the gastrointestinal tract of birds, it is widely present in birds’ excreta [35]. Infection with these gram-negative bacteria is the main cause of diarrhea. It has been found that contamination of poultry litter with *Escherichia coli* may dominate the litter and vary from 3.3 to above 7 log CFU/g of litter, which is in line with our results. During the experiment we observed relatively high (6–9 log CFU/g) contamination of *Escherichia* sp. in both experimental cycles. The material P1 was significantly less contaminated (a difference of 1–2 logs CFU/g) compared to straw chaff in both experimental cycles at day 7, although the effect was not maintained in the following days (See Figure 5). These results go beyond previous reports, showing that a significant reduction of *Escherichia coli* can be reached, not only through application of chemicals, such as alum, which reduced the *E. coli* count by 2 logs CFU/g (application of alum on sawdust bedding from commercial broiler house after 4 flock cycles [78]). Still, even more significant than in our study, were reported in prior studies that investigated the heat treatment (35–65 °C) of litter collected from poultry houses (mainly rice hulls), which resulted in a 2–6 log reduction of the initial *E. coli* concentration in lab scale conditions [51,79]. However, it should be noted that the advantage of including parts of medicinal plants in the bedding materials is that they potentially offer antimicrobial properties during the whole production process, while other approaches, e.g., application of alum or heat treatment, can be applied only after the finished production cycle. Still, our results suggest that the application of the studied materials does not replace the use of other practices. However, it may complement them.

#### 4.3.4. *Listeria* sp.

The major reservoir for antibiotic resistance genes in poultry litter are Gram-positive bacteria that represent >85% of the microbial community compared with Gram-negative ones that comprise <2% of such ecosystems [12]. *Listeria* belongs to the Gram-positive species. It is found in soil, sewage, birds’ feces, and surface water [80]. Even though several prior studies have confirmed the incidence of significant counts of *Listeria* sp., including *Listeria monocytogenes*, in poultry beddings [81,82], we are the first to report on the dynamics of this bacteria development over the poultry bedding exploitation. Our results suggest that *Listeria* sp. growth dynamics is similar to the total aerobic microbial flora and *Staphylococci* development. The level of contamination was estimated from 6.65 to 8.5 log CFU/g during the experimental cycles. A promising finding was that less *Listeria* sp. was found after 14 days of use in all tested bedding materials than in straw chaff in the second production cycle. This could be explained by the fact that savory essential oil works effectively against *Listeria* sp. [83]. Still, further research on the antimicrobial effectiveness of herbs in bedding materials is needed to explain the mechanism of this observation. The main limitation in answering the question of what concentration of herbs is needed to significantly reduce the growth of *Listeria* sp. bacteria during the entire production cycle is the limited knowledge on the release of essential oils during the bedding material use.

#### 4.3.5. *Salmonella* sp.

*Salmonella enterica* is one of the pathogens that contaminate feedstuff, infect chickens, and then lead to disease in humans [84]. Though not in the whole experimental cycles, we have observed the presence of this genera in each bedding material. This is not consistent with the findings of prior research, where *Salmonella* was found only in 50% of wood shavings samples, in 30% of peat samples, and 17% of corn silage samples [17]. Our results do not repeat the observations concerning *Salmonella* in the chopped straw samples, where no incidence of this bacteria were reported [17]. During our study, no *Salmonella* sp. was found, apart from in the P5 material after 7 days of bedding use in the second experimental cycle.

In samples taken from poultry houses in the United States, *Salmonella* contamination in bedding material occurred during each of the three tested dates (pre-flock = no chickens; early flock = 1–3 weeks; late-flock = 4–6 weeks) and was in a range of 1–4 log CFU/g [75]. Our findings are in line with these results. High contamination of the tested materials with *Salmonella* sp. (1–7 log CFU/g) in both of our experimental cycles were observed. In the first cycle, a lower contamination with *Salmonella* sp. was determined for P4 and P5 materials compared to the straw pellet (4.21 vs. 5.48 log CFU/g). The use of the studied materials could be regarded as a measure of limiting the application of certain chemical additives as *Salmonella* growth inhibitors, at least to some extent, e.g., the hydrated lime, which has been demonstrated as a viable measure for reducing *Salmonella* in chicken litter [85].

#### 4.3.6. *Candida* sp.

Similar to the case of *Listeria* sp., to the best of our knowledge the presented results are the first reports on the dynamics of *Candida* sp. development over the exploitation of poultry bedding materials. Pathogenic *Candida albicans* are ubiquitous in animal housing facilities [78]. Acidifying the bedding material was reported to have no selection effect for this fungi [78]. Similar to these findings, we reported significant *Candida* sp. as well as yeast and mould decreases in the bedding materials with increasing pH values.

### 4.4. Limitations and Future Research

Our research may have suffered from the bias arising from the allocation sequence generation. It was not fully random, since materials were placed in the compartments in line with their consecutive numbers. Still, our judgement of the risk of bias arising from the randomisation process is that it was low, so the reported significant differences between materials were genuine. Our judgement is based on the following arguments: (1) no contact between animals or materials from different compartments was possible; (2) the conditions of air temperature and humidity in all compartments were uniform; (3) the allocation of animals to compartments was random and balanced. To minimise the risk of bias arising from the randomisation process, future research could use computer-generated random numbers for the allocation sequence generation.

On several days of our experiments, the studied materials comprising parts of medicinal plants demonstrated a higher water absorption capacity than straw chaff, which is one of the most popular conventional bedding materials used in poultry production. Therefore, all of them are promising solutions, deserving further studies. The antimicrobial effects of using materials comprising parts of medicinal plants have been also observed on several days, e.g., on day 14 of both experimental cycles, the count of *Staphylococcus* sp. in P4 was significantly lower than in the straw pellets. This suggests that P4 could be regarded as a more adequate material for improving the hygienic conditions of younger animals, or for increasing the biosecurity of poultry farms struggling with infections caused by *Staphylococci*. Further research is needed to investigate the potential relations between antimicrobial effects and an increased mass share of raw materials from medicinal plants in the animal bedding.

## 5. Conclusions

In the present study we demonstrated that the use of materials comprising parts of medicinal plants (*Satureja hortensis*, *Origanum vulgare*, *Melissa officinalis*, *Salvia officinalis*, and *Thymus vulgaris)* can lead to:

1. a lower total count of mesophilic aerobic bacteria in poultry bedding,
2. a lower total count of yeasts and moulds in poultry bedding,
3. a lower presumptive count of *Staphylococcus* sp., *Escherichia* sp., *Listeria* sp., *Salmonella* sp., and *Candida* sp. in poultry bedding.

However, the occurrence and strength of the antimicrobial effects vary over time (42 days of broiler production) and depend on the material composition. These effects are likely to be caused not only by the essential oils of medicinal plants, but also the high water absorption capacity of the studied materials and the low microbial contamination of materials prior their exploitation. Both of these features are due to the materials production technique (pelletisation).

The investigated materials offer potential advantages over other materials and practices of ensuring good hygienic conditions in poultry production, such as the application of alum or heat treatment to litter, which can be applied only after the finished production cycle. On the other hand, the use of medicinal plants in the materials may increase bedding material costs. To improve the economics of materials production, an increased share of waste herbal materials (e.g., stems), as ingredients of beddings should be studied.

## 6. Patents

The work reported in this manuscript has resulted in the patent application for the invention titled “Bedding material and the method of producing bedding material” submitted to the Polish Patent Office (P. 439186).

## Figures and Tables

**Table 1:** Bedding materials compositions.

Material Type	Material Composition					
	Ingredient 1 (I1)	I1 % (*w*/*w*)	Ingredient 2 (I2)	I2 % (*w*/*w*)	Ingredient 3 (I3)	I3 % (*w*/*w*)
P1	triticale straw	70	*Origanum vulgare* herb	24	*Satureja hortensis* herb	6
P2	triticale straw	70	*Satureja hortensis* herb	18	*Origanum vulgare* stems	12
P3	triticale straw	70	*Satureja hortensis* herb	18	*Melissa officinalis* stems	12
P4	triticale straw	70	*Satureja hortensis* herb	18	*Salvia officinalis* stems	12
P5	triticale straw	70	*Satureja hortensis* herb	18	*Thymus vulgaris* stems	12
P6	triticale straw	70	*Origanum vulgare* herb	24	*Thymus vulgaris* leaves	6
Straw pellet	triticale straw	100	-	-	-	-
Straw chaff	triticale straw	100	-	-	-	-

**Table 2:** Moisture and essential oil content, pH, water absorption capacity and total count of aerobic mesophilic bacteria in the tested bedding materials prior their exploitation.

Bedding Material	Moisture Content	Essential Oil Content in Input Ingredients before Processing [mL/100 g a.d.m.]	Essential Oil Content in Material [mL/100 g a.d.m.]	Water Absorption Capacity [%]	pH [–]	Total Count of Aerobic Mesophilic Bacteria [log CFU/g]
P1	12.41 ± 0.06 ^d^	0.27 ± 0.01 ^c^	0.09 ± 0.01 ^d^	377.83 ± 10.12 ^a^	6.28 ± 0.02 ^d^	5.88 ± 0.20 ^b^
P2	14.37 ± 0.02 ^b^	0.71 ± 0.02 ^a^	0.30 ± 0.01 ^b^	242.43 ± 13.20 ^d^	6.46 ± 0.02 ^c^	5.13 ± 0.12 ^bc^
P3	14.54 ± 0.02 ^b^	0.71 ± 0.02 ^a^	0.34 ± 0.04 ^ab^	346.43 ± 11.25 ^b^	6.44 ± 0.01 ^c^	5.30 ± 0.27 ^bc^
P4	12.96 ± 0.02 ^c^	0.72 ± 0.02 ^a^	0.35 ± 0.02 ^a^	375.28 ± 10.10 ^a^	6.50 ± 0.01 ^b^	5.36 ± 0.55 ^bc^
P5	12.04 ± 0.08 ^d^	0.53 ± 0.01 ^b^	0.23 ± 0.01 ^c^	392.27 ± 12.30 ^a^	6.55 ± 0.02 ^a^	7.20 ± 0.01 ^a^
P6	11.51 ± 0.09 ^e^	0.19 ± 0.02 ^d^	0.09 ± 0.01 ^d^	390.55 ± 11.60 ^a^	6.44 ± 0.02 ^c^	5.00 ± 0.04 ^c^
Straw pellet	12.99 ± 0.11 ^c^	-	-	300.58 ± 10.30 ^c^	6.51 ± 0.03 ^b^	5.51 ± 0.35 ^bc^
Straw chaff	16.87 ± 0.09 ^a^	-	-	277.97 ± 16.02 ^c^	6.20 ± 0.03 ^e^	7.01 ± 0.08 ^a^

Note: All values are means of three independent replicates ± SD. Within each column, means with a common lowercase letter are not significantly different by the Tukey test at the 5% level of significance.

**Table 3:** Changes in the pH of bedding materials during their exploitation—1st experimental cycle.

Bedding Material	pH after a Given Period of Use under Operational Conditions					
	Day 7	Day 14	Day 21	Day 28	Day 35	Day 42
P1	6.33 ± 0.00 ^bc^	6.40 ± 0.04 ^ab^	7.02 ± 0.05 ^a^	7.48 ± 0.05 ^a^	7.19 ± 0.11 ^a^	6.95 ± 0.24 ^a^
P2	6.47 ± 0.01 ^a^	6.30 ± 0.13 ^b^	7.07 ± 0.01 ^a^	7.34 ± 0.05 ^a^	7.19 ± 0.07 ^a^	6.96 ± 0.15 ^a^
P3	6.44 ± 0.03 ^ab^	6.33 ± 0.11 ^ab^	7.04 ± 0.08 ^a^	7.13 ± 0.08 ^a^	7.31 ± 0.06 ^a^	6.93 ± 0.09 ^a^
P4	6.41 ± 0.01 ^ab^	6.30 ± 0.18 ^b^	6.83 ± 0.01 ^a^	7.24 ± 0.24 ^a^	7.38 ± 0.08 ^a^	6.72 ± 0.28 ^a^
P5	6.39 ± 0.04 ^abc^	6.36 ± 0.04 ^ab^	7.01 ± 0.05 ^a^	7.34 ± 0.11 ^a^	7.25 ± 0.14 ^a^	7.06 ± 0.10 ^a^
P6	6.49 ± 0.03 ^a^	6.42 ± 0.04 ^ab^	6.93 ± 0.07 ^a^	7.44 ± 0.04 ^a^	7.17 ± 0.02 ^a^	6.86 ± 0.07 ^a^
Straw pellet	6.48 ± 0.09 ^a^	6.65 ± 0.03 ^a^	7.04 ± 0.02 ^a^	7.32 ± 0.10 ^a^	7.40 ± 0.03 ^a^	6.87 ± 0.04 ^a^
Straw chaff	6.26 ± 0.03 ^c^	6.54 ± 0.10 ^ab^	6.98 ± 0.20 ^a^	7.22 ± 0.17 ^a^	7.19 ± 0.00 ^a^	7.00 ± 0.07 ^a^
Mean	6.41 ± 0.01 ^C^	6.41 ± 0.01 ^C^	6.99 ± 0.04 ^B^	7.32 ± 0.01 ^A^	7.26 ± 0.04 ^A^	6.92 ± 0.05 ^B^

Note: All values for P1–P6 as well as straw pellet and chaff are means of three independent replicates ± SD. The mean in the last row is mean-of-means for all materials ± SD. For each day, means with a common lowercase letter are not significantly different by the Tukey test at the 5% level of significance. For different days, means-of-means with a common uppercase letter are not significantly different by the Tukey test at the 5% level of significance.

**Table 4:** Changes in the pH of bedding materials during their exploitation—2nd experimental cycle.

Bedding Material	pH after a Given Period of Use under Operational Conditions					
	Day 7	Day 14	Day 21	Day 28	Day 35	Day 42
P1	6.34 ± 0.02 ^ab^	6.28 ± 0.00 ^b^	6.65 ± 0.09 ^a^	7.56 ± 0.04 ^a^	7.57 ± 0.09 ^a^	7.49 ± 0.10 ^a^
P2	6.43 ± 0.00 ^a^	6.40 ± 0.04 ^ab^	6.77 ± 0.06 ^a^	7.59 ± 0.07 ^a^	7.45 ± 0.07 ^a^	7.43 ± 0.05 ^a^
P3	6.35 ± 0.03 ^ab^	6.28 ± 0.03 ^b^	6.51 ± 0.01 ^a^	7.42 ± 0.02 ^a^	7.45 ± 0.08 ^a^	7.47 ± 0.10 ^a^
P4	6.35 ± 0.04 ^ab^	6.36 ± 0.05 ^ab^	6.69 ± 0.04 ^a^	7.60 ± 0.14 ^a^	7.21 ± 0.11 ^a^	7.15 ± 0.16 ^a^
P5	6.35 ± 0.05 ^ab^	6.17 ± 0.12 ^b^	6.58 ± 0.07 ^a^	7.59 ± 0.25 ^a^	7.37 ± 0.18 ^a^	7.56 ± 0.33 ^a^
P6	6.36 ± 0.01 ^ab^	6.36 ± 0.05 ^ab^	6.64 ± 0.02 ^a^	7.62 ± 0.06 ^a^	7.22 ± 0.29 ^a^	7.45 ± 0.07 ^a^
Straw pellet	6.45 ± 0.06 ^a^	6.35 ± 0.03 ^ab^	6.70 ± 0.12 ^a^	7.61 ± 0.07 ^a^	7.31 ± 0.09 ^a^	7.50 ± 0.12 ^a^
Straw chaff	6.21 ± 0.11 ^b^	6.55 ± 0.13 ^a^	6.67 ± 0.16 ^a^	7.30 ± 0.28 ^a^	7.12 ± 0.20 ^a^	7.47 ± 0.18 ^a^
Mean	6.35 ± 0.01 ^D^	6.35 ± 0.01 ^D^	6.65 ± 0.02 ^C^	7.54 ± 0.09 ^A^	7.34 ± 0.02 ^B^	7.44 ± 0.04 ^AB^

Note: All values for P1–P6 as well as straw pellet and chaff are means of three independent replicates ± SD. The mean in the last row is mean-of-means for all materials ± SD. For each day, means with a common lowercase letter are not significantly different by the Tukey test at the 5% level of significance. For different days, means-of-means with a common uppercase letter are not significantly different by the Tukey test at the 5% level of significance.

**Table 5:** Changes in the count of mesophilic aerobic bacteria in the bedding material during use under operational conditions—1st experimental cycle.

Bedding Material	Log CFU/g after a Given Period of Use under Operational Conditions					
	Day 7	Day 14	Day 21	Day 28	Day 35	Day 42
P1	8.22 ± 0.07 ^c^	9.32 ± 0.21 ^bc^	11.11 ± 0.64 ^a^	15.44 ± 0.15 ^a^	15.62 ± 0.04 ^a^	15.68 ± 0.32 ^a^
P2	8.49 ± 0.10 ^bc^	9.61 ± 0.04 ^abc^	11.20 ± 0.23 ^a^	15.57 ± 0.30 ^a^	15.53 ± 0.11 ^a^	15.00 ± 0.06 ^a^
P3	9.38 ± 0.22 ^a^	9.21 ± 0.08 ^c^	11.09 ± 0.12 ^a^	15.71 ± 0.04 ^a^	15.69 ± 0.12 ^a^	15.50 ± 0.47 ^a^
P4	8.64 ± 0.22 ^bc^	9.33 ± 0.24 ^bc^	10.83 ± 0.16 ^a^	15.48 ± 0.23 ^a^	15.91 ± 0.24 ^a^	14.95 ± 0.43 ^a^
P5	8.99 ± 0.44 ^ab^	9.40 ± 0.14 ^bc^	11.07 ± 0.59 ^a^	15.74 ± 0.34 ^a^	15.75 ± 0.26 ^a^	15.31 ± 0.27 ^a^
P6	9.04 ± 0.13 ^ab^	9.61 ± 0.15 ^abc^	11.18 ± 0.36 ^a^	15.54 ± 0.33 ^a^	15.64 ± 0.10 ^a^	15.23 ± 0.28 ^a^
Straw pellet	9.63 ± 0.06 ^a^	9.95 ± 0.05 ^a^	11.47 ± 0.23 ^a^	15.39 ± 0.09 ^a^	15.50 ± 0.44 ^a^	15.08 ± 0.25 ^a^
Straw chaff	9.43 ± 0.13 ^a^	9.78 ± 0.12 ^ab^	12.05 ± 0.11 ^a^	15.47 ± 0.33 ^a^	15.40 ± 0.38 ^a^	14.99 ± 0.38 ^a^
Mean	8.98 ± 0.02 ^E^	9.53 ± 0.06 ^D^	11.25 ± 0.13 ^C^	15.54 ± 0.05 ^A^	15.63 ± 0.06 ^A^	15.22 ± 0.17 ^B^

Note: All values for P1–P6, as well as straw pellet and chaff, are means of three independent replicates ± SD. The mean in the last row is mean-of-means for all materials ± SD. For each day, means with a common lowercase letter are not significantly different by the Tukey test at the 5% level of significance. For different days, means-of-means with a common uppercase letter are not significantly different by the Tukey test at the 5% level of significance.

**Table 6:** Changes in the count of mesophilic aerobic bacteria in the bedding material during use under operational conditions—2nd experimental cycle.

Bedding Material	Log CFU/g after a Given Period of Use under Operational Conditions					
	Day 7	Day 14	Day 21	Day 28	Day 35	Day 42
P1	7.73 ± 0.56 ^b^	9.47 ± 0.13 ^c^	10.01 ± 0.09 ^b^	11.01 ± 0.46 ^a^	14.36 ± 0.09 ^a^	12.75 ± 0.14 ^b^
P2	8.87 ± 0.02 ^a^	10.18 ± 0.13 ^ab^	10.39 ± 0.10 ^ab^	11.40 ± 0.34 ^a^	14.21 ± 0.50 ^a^	12.92 ± 0.32 ^b^
P3	8.77 ± 0.42 ^ab^	9.51 ± 0.15 ^c^	10.17 ± 0.05 ^ab^	10.79 ± 0.36 ^a^	11.86 ± 0.11 ^d^	12.78 ± 0.45 ^b^
P4	8.34 ± 0.44 ^ab^	9.94 ± 0.30 ^abc^	10.51 ± 0.12 ^ab^	11.46 ± 0.54 ^a^	12.28 ± 0.54 ^cd^	10.95 ± 0.02 ^c^
P5	7.94 ± 0.12 ^ab^	9.79 ± 0.13 ^bc^	10.42 ± 0.24 ^ab^	11.44 ± 0.21 ^a^	12.04 ± 0.30 ^cd^	13.36 ± 0.52 ^ab^
P6	8.61 ± 0.20 ^ab^	9.61 ± 0.23 ^bc^	10.55 ± 0.16 ^a^	10.75 ± 0.25 ^a^	12.29 ± 0.24 ^cd^	13.10 ± 0.44 ^ab^
Straw pellet	8.77 ± 0.25 ^ab^	10.03 ± 0.17 ^abc^	10.19 ± 0.25 ^ab^	11.25 ± 0.35 ^a^	13.02 ± 0.17 ^bc^	14.12 ± 0.25 ^a^
Straw chaff	8.99 ± 0.16 ^a^	10.46 ± 0.04 ^a^	10.29 ± 0.10 ^ab^	11.66 ± 0.07 ^a^	13.78 ± 0.15 ^ab^	13.19 ± 0.34 ^ab^
Mean	8.50 ± 0.17 ^F^	9.87 ± 0.08 ^E^	10.32 ± 0.03 ^D^	11.22 ± 0.11 ^C^	12.98 ± 0.11 ^A^	12.27 ± 0.09 ^B^

Note: All values for P1–P6, as well as straw pellet and chaff, are means of three independent replicates ± SD. The mean in the last row is mean-of-means for all materials ± SD. For each day, means with a common lowercase letter are not significantly different by the Tukey test at the 5% level of significance. For different days, means-of-means with a common uppercase letter are not significantly different by the Tukey test at the 5% level of significance.

**Table 7:** Changes in the count of total yeast and moulds in the bedding material during use under operating conditions—1st experimental cycle.

Bedding Material	Log CFU/g after a Given Period of Use under Operational Conditions					
	Day 7	Day 14	Day 21	Day 28	Day 35	Day 42
P1	5.97 ± 0.17 ^bcd^	7.93 ± 0.11 ^a^	8.25 ± 0.03 ^b^	7.69 ± 0.14 ^a^	6.64 ± 0.34 ^a^	6.29 ± 0.10 ^a^
P2	6.56 ± 0.37 ^abc^	8.11 ± 0.09 ^a^	8.32 ± 0.14 ^ab^	7.72 ± 0.22 ^a^	6.39 ± 0.33 ^a^	6.24 ± 0.05 ^a^
P3	6.96 ± 0.22 ^a^	7.23 ± 0.70 ^a^	8.43 ± 0.15 ^ab^	7.93 ± 0.24 ^a^	6.67 ± 0.11 ^a^	5.16 ± 0.38 ^b^
P4	5.30 ± 0.23 ^d^	7.43 ± 0.52 ^a^	8.25 ± 0.17 ^a^	7.96 ± 0.14 ^a^	6.54 ± 0.17 ^a^	5.55 ± 0.46 ^ab^
P5	7.08 ± 0.10 ^a^	8.00 ± 0.03 ^a^	8.18 ± 0.11 ^ab^	7.73 ± 0.20 ^a^	6.39 ± 0.19 ^a^	5.55 ± 0.04 ^ab^
P6	5.78 ± 0.15 ^cd^	7.97 ± 0.11 ^a^	7.96 ± 0.19 ^ab^	7.77 ± 0.10 ^a^	6.46 ± 0.13 ^a^	5.84 ± 0.34 ^ab^
Straw pellet	6.50 ± 0.35 ^abc^	7.89 ± 0.14 ^a^	8.03 ± 0.02 ^ab^	6.98 ± 0.04 ^b^	6.44 ± 0.18 ^a^	5.91 ± 0.04 ^ab^
Straw chaff	6.87 ± 0.37 ^ab^	8.02 ± 0.12 ^a^	8.39 ± 0.07 ^ab^	7.40 ± 0.14 ^ab^	6.74 ± 0.07 ^a^	5.47 ± 0.35 ^ab^
Mean	6.38 ± 0.06 ^C^	7.82 ± 0.05 ^B^	8.23 ± 0.03 ^A^	7.65 ± 0.03 ^B^	6.54 ± 0.14 ^C^	5.75 ± 0.14 ^D^

Note: All values for P1–P6, as well as straw pellet and chaff, are means of three independent replicates ± SD. The mean in the last row is mean-of-means for all materials ± SD. For each day, means with a common lowercase letter are not significantly different by the Tukey test at the 5% level of significance. For different days, means-of-means with a common uppercase letter are not significantly different by the Tukey test at the 5% level of significance.

**Table 8:** Changes in the count of total yeast and moulds in the bedding material during use under operating conditions—2nd experimental cycle.

Bedding Material	Log CFU/g after a Given Period of Use under Operational Conditions					
	Day 7	Day 14	Day 21	Day 28	Day 35	Day 42
P1	6.40 ± 0.65 ^ab^	7.74 ± 0.15 ^b^	7.84 ± 0.08 ^abc^	6.38 ± 0.33 ^a^	6.23 ± 0.07 ^a^	6.41 ± 0.08 ^a^
P2	7.62 ± 0.06 ^a^	7.72 ± 0.06 ^b^	7.60 ± 0.02 ^bc^	6.10 ± 0.23 ^a^	6.62 ± 0.40 ^a^	6.06 ± 0.20 ^ab^
P3	7.05 ± 0.55 ^a^	7.54 ± 0.14 ^b^	7.55 ± 0.12 ^c^	6.27 ± 0.15 ^a^	6.02 ± 0.08 ^a^	5.95 ± 0.29 ^abc^
P4	6.69 ± 0.03 ^ab^	7.60 ± 0.05 ^b^	7.96 ± 0.17 ^ab^	6.21 ± 0.16 ^a^	6.33 ± 0.03 ^a^	5.92 ± 0.18 ^abcd^
P5	5.12 ± 0.51 ^b^	7.82 ± 0.11 ^b^	8.10 ± 0.13 ^a^	6.65 ± 0.40 ^a^	6.03 ± 0.17 ^a^	5.45 ± 0.21 ^cd^
P6	7.36 ± 0.03 ^a^	7.69 ± 0.04 ^b^	7.92 ± 0.08 ^abc^	5.98 ± 0.01 ^a^	6.13 ± 0.21 ^a^	5.48 ± 0.07 ^bcd^
Straw pellet	6.26 ± 0.82 ^ab^	7.52 ± 0.14 ^b^	7.76 ± 0.11 ^abc^	5.73 ± 0.42 ^a^	6.06 ± 0.15 ^a^	6.21 ± 0.16 ^a^
Straw chaff	7.04 ± 0.16 ^a^	8.62 ± 0.07 ^a^	8.00 ± 0.09 ^a^	6.58 ± 0.17 ^a^	6.30 ± 0.18 ^a^	5.32 ± 0.08 ^d^
Mean	6.69 ± 0.02 ^B^	7.78 ± 0.03 ^A^	7.84 ± 0.04 ^A^	6.24 ± 0.09 ^C^	6.22 ± 0.04 ^C^	5.85 ± 0.11 ^D^

Note: All values for P1–P6, as well as straw pellet and chaff, are means of three independent replicates ± SD. The mean in the last row is mean-of-means for all materials ± SD. For each day, means with a common lowercase letter are not significantly different by the Tukey test at the 5% level of significance. For different days, means-of-means with a common uppercase letter are not significantly different by the Tukey test at the 5% level of significance.

## Data Availability

The data that support the findings of this study are available from the corresponding author, M.S.-A., upon request.
